# Economical and scalable synthesis of 6-amino-2-cyanobenzothiazole

**DOI:** 10.3762/bjoc.12.189

**Published:** 2016-09-13

**Authors:** Jacob R Hauser, Hester A Beard, Mary E Bayana, Katherine E Jolley, Stuart L Warriner, Robin S Bon

**Affiliations:** 1 School of Chemistry, University of Leeds; 2 Astbury Centre for Structural Molecular Biology; 3 Institute of Process Research and Development,; 4 Leeds Institute of Cardiovascular and Metabolic Medicine, LIGHT Laboratories, University of Leeds, Leeds LS2 9JT, UK

**Keywords:** ACBT, cyanation, 2-cyanobenzothiazoles, DABCO, luciferins, organocatalysis

## Abstract

2-Cyanobenzothiazoles (CBTs) are useful building blocks for: 1) luciferin derivatives for bioluminescent imaging; and 2) handles for bioorthogonal ligations. A particularly versatile CBT is 6-amino-2-cyanobenzothiazole (ACBT), which has an amine handle for straight-forward derivatisation. Here we present an economical and scalable synthesis of ACBT based on a cyanation catalysed by 1,4-diazabicyclo[2.2.2]octane (DABCO), and discuss its advantages for scale-up over previously reported routes.

## Introduction

**Scheme 1:**
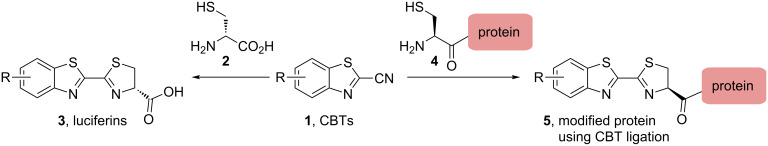
Functionalised CBTs **1** can be used for the synthesis of luciferins **3** and for bioorthogonal ligations such as the site-specific labelling/immobilisation of proteins **4**.

Functionalised 2-cyanobenzothiazoles (CBTs, **1**) are key building blocks for the synthesis of luciferins **3** [1–3], substrates of natural and engineered firefly luciferases that are widely used for bioluminescence imaging (BLI) [4–5]. The typical preparation of luciferins **3** involves straightforward condensation of CBTs **1** with D-cysteine (**2**) (Scheme 1). For BLI applications, luciferins are often generated in vivo from CBTs [4–7], which are easier to modify and handle due to their higher stability, cell permeability, and reactivity than the full luciferin scaffolds [2,5,8].

Even in the presence of other thiols and amines, CBTs react rapidly and selectively with 1,2-aminothiols under physiological conditions. This selective and bioorthogonal reactivity of CBTs has been exploited in the development of the CBT ligation as a useful and fast bioorthogonal reaction (*k* ≈ 10 M^−1^s^−1^) [9] for site-specific labelling or immobilisation of proteins **4**, either at an N-terminal cysteine residue or at a 1,2-aminothiol group incorporated into a non-natural amino acid (Scheme 1) [10–11]. In addition, CBT derivatives have been used for the synthesis of polymeric nanostructures in cellulo [12–13]*.*

The precursor of D-aminoluciferin, 6-amino-2-cyanobenzothiazole (ACBT, **8**), is an attractive building block for BLI probes and for handles for CBT ligations, because of the ease of derivatisation of its amino group. Like other functionalised CBTs, ACBT is available from commercial suppliers, but expensive. Thus, the development of an economical and scalable synthesis of ACBT would enable studies that require larger amounts of this building block.

**Scheme 2:**
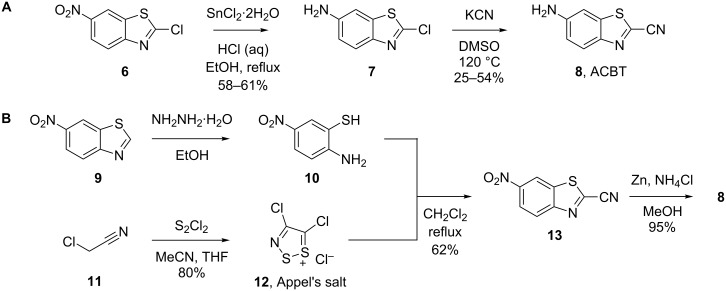
Reported synthetic routes to ACBT **8**: A) Original route reported by Takakura et al. [14] and Wang et al. [15]. B) The improved route reported by McCutcheon et al. [7].

The traditional synthesis of ACBT **8** involves the cyanation of 6-amino-2-chlorobenzothiazole (**7**) with an excess of potassium cyanide (KCN) in hot DMSO under dilute conditions (Scheme 2A) [14–15]. However, this reaction is sluggish (partly because of the poor solubility of KCN in DMSO), requires elaborate extractive workups for the removal of DMSO (persistent traces of which are detrimental to purification) and gives low and variable yields (typically 30–50% in our hands). In addition, because DMSO can quickly penetrate the skin, working with KCN in DMSO requires substantial safety measures. Therefore, this route does not allow straightforward scale-up under laboratory conditions. Prescher and co-workers reported an alternative synthesis of ACBT that avoids the use of cyanide (Scheme 2B) [7]. This route makes use of 4,5-dichloro-1,2,3-dithiazolium chloride (Appel’s salt, **12**), and variations of the procedure have enabled the synthesis of different CBT derivatives, some of which on multi-gram scale [6–7,16–17]. However, Appel’s salt is relatively expensive and available from a limited number of suppliers, and its synthesis requires the use of the highly toxic reagent sulfur monochloride [18].

Here, we report an alternative, practical and economical procedure for the preparation of ACBT **8**. Our route involves a mild, organocatalytic cyanation. In addition, the use of filtrations and crystallisations for purification, in combination with control of reaction rate and heat output in the cyanation step, makes this procedure readily scalable.

## Results and Discussion

In order to install the cyano group of ACBT **8** under mild conditions, catalytic cyanation procedures were considered. Initial attempts to use the palladium or copper-catalysed cyanation of 6-amino-2-halobenzothiazoles with potassium hexaferricyanide – a slow-releasing cyanide source – were unsuccessful. Instead, a report on the use of 1,4-diazabicyclo[2.2.2]octane (DABCO) as a catalyst for the cyanation of heteroaryl halides [19] inspired us to explore the DABCO-catalysed cyanation of 2-chlorobenzothiazoles.

Indeed, the treatment of 2-chloro-6-nitrobenzothiazole (**6**) with DABCO (15 mol %) and sodium cyanide (NaCN, 1.05 equiv) in DMSO/water 1:1, at room temperature, resulted in full conversion to 2-cyano-6-nitrobenzothiazole (**13**, Table 1), a known precursor of ACBT **8** [7]. Any unreacted cyanide in the reaction mixture was safely quenched by the addition of an iron(III) chloride solution, and pure ACBT **8** was isolated in 90% yield after (extensive) extractive work-up and flash chromatography. No conversion of **6** to **13** was observed in the absence of DABCO, which demonstrates the essential role of DABCO as a cyanation catalyst under these conditions. In addition, when the more electron rich 6-amino-2-chlorobenzothiazole (**7**) was subjected to the DABCO-catalysed cyanation conditions, only starting material was isolated, even upon heating the reaction mixture to 100 °C (not shown). This suggests that, although the cyanation may be adaptable to other 2-halobenzothiazole derivatives, electron-deficient substrates may be required for the reactions to proceed under mild conditions.

**Table 1:** DABCO-catalysed cyanation of **6**: solvent studies.^a^

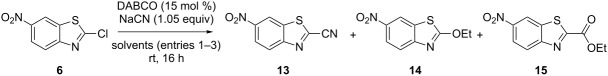				

entry	solvent(s)	conversion of **6**^b^	ratio **13**:**14**:**15**^c^	yield **13**^d^
				
1	DMSO/H_2_O 1:1	100%	100:0:0	90%
2	EtOH	100%	17:32:51	ND
3	MeCN/H_2_O 10:1	100%	100:0:0	75%

^a^Reactions were performed using 200 mg **6** in 20 mL solvent. ^b^Conversion of **6** was determined by LC–ESIMS. ^c^Identities of **13**–**15** in the reaction mixtures were confirmed by ^1^H NMR and LC–MS and product ratios were determined by ^1^H NMR after work-up. ^d^Yields were determined after purification; ND = not determined.

In view of scale-up of the DABCO-catalysed cyanation of **6**, an alternative solvent (mixture) was sought in order to eliminate safety issues and work-up problems resulting from the use of DMSO. An initial attempt to run the reaction in water failed because of the low aqueous solubility of **6** – resulting in a biphasic reaction mixture – and the slow hydrolysis of **6** under the reaction conditions according to LC–MS (not shown). The conversion of **6** was complete in ethanol. However, in addition to the expected cyano compound **13**, significant amounts of side products **14** (resulting from ethanolysis of **6**) and **15** (resulting from ethanolysis of **13** and subsequent hydrolysis of the intermediate imidate) were formed as well (Table 1, entry 2; Supporting Information File, Figure S7). In contrast, the use of acetonitrile (with a small volume of water to facilitate dissolution/addition of NaCN) resulted in full conversion to **13**, and pure **13** which was isolated in 75% yield after quenching with iron(III) chloride solution, extraction and simple filtration through a silica plug (Table 1, entry 3). The straightforward work-up/purification procedure – in the absence of DMSO as a co-solvent – outweighed the slightly reduced reaction yield on this scale.

**Figure 1:**
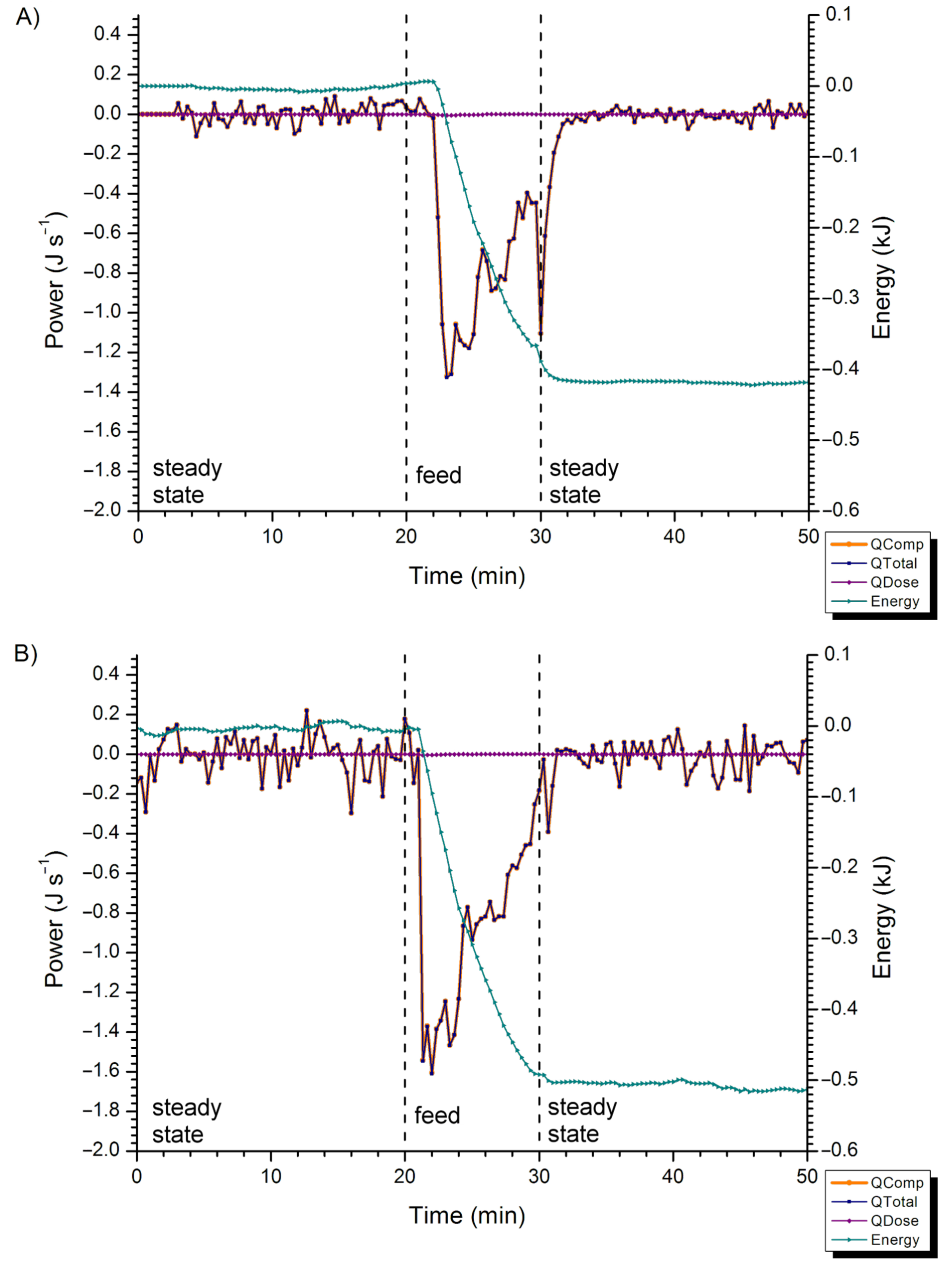
Calorimeter traces for the addition of aqueous NaCN (A) or water (B) to a solution of **6** and DABCO in acetonitrile. QComp: compensatory power; QTotal: total power; QDose: power generated due to the temperature and heat capacity of the feed; energy: heat energy. Because QDose ≈ 0 over the course of the experiment, traces for QComp (orange) and QTotal (blue) overlap.

**Scheme 3:**
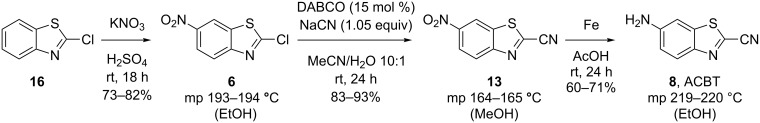
Scale-up of ACBT synthesis.

For scale-up of the DABCO-catalysed cyanation, **6** was synthesised by straight-forward nitration of the significantly cheaper starting material 2-chlorobenzothiazole (**16**) [15], and isolated in 82% yield after crystallisation from ethanol (Scheme 3). Recrystallisation of **16** from acetonitrile required only 25% of the solvent volume, but resulted in a slightly lower yield (73%). In view of potential dangers involved in the use of NaCN on large scale, a calorimetric analysis was conducted to monitor the heat output of the cyanation reaction. In this experiment, aqueous NaCN (49 mg in 2 mL) was slowly added to a solution of **6** (200 mg) and DABCO (15 mol %) in acetonitrile (20 mL; compound **6** dissolves in acetonitrile only upon addition of DABCO). The calorimeter jacket temperature was set at 1 °C, and power compensation by an internal heater coil was used to maintain the reactor temperature at 21 °C. The reaction resulted in an endotherm (Figure 1A). The total power released or consumed by the reaction (QTotal) was calculated by subtracting the power generated due to the temperature and specific heat capacity of the feed (QDose) from the compensatory power supplied by the internal heater coil (QComp). Because QDose remained at zero throughout the reaction, the traces for QComp and QTotal overlap in Figure 1A. From QTotal, an energy consumption of 0.42 kJ was determined for the addition of 1 mmol of NaCN in 2 mL of water. A control experiment, in which only water was added to **6** and DABCO in acetonitrile, showed an endotherm of 0.51 kJ, corresponding to a calculated heat of mixing of water into the reaction mixture of Δ*H* = 4.57 kJ mol^−1^ (see Supporting Information File 1). These observations complicated accurate quantification of the heat of reaction (because of different properties of the feeds in the two experiments). The difference in endotherms between the cyanation reaction (Figure 1A) and the control experiment (Figure 1B) suggests the overall DABCO-catalysed reaction of **6** and NaCN is exothermic, but that the endothermic addition of water to the acetonitrile solution outweighs this exotherm under the experimental conditions. Our results suggest that a slow addition of a dilute aqueous NaCN solution is a safe method for scale-up of the cyanation reaction, provided the concentration of the solution allows an overall reaction endotherm to be maintained to prevent potential thermal runaway upon scale-up.

The DABCO-catalysed cyanation reaction was scaled up in two steps: using 2 g and then 10 g of substrate **6** (Scheme 3). After quenching with iron(III) chloride solution, extractive work-up and filtration through a short silica plug, product **13** was isolated in 83–93% yield. A sample of **13** for melting point determination was obtained by recrystallisation from methanol. The synthesis of ACBT **8** was completed by reduction of the nitro group in **13** (Scheme 3). In our hands, the use of iron powder in acetic acid proved more practical than the previously reported procedures with zinc powder and ammonium chloride [7]. It resulted in more consistent yields, an easier work-up, and no persistent contamination of **8** with nitroso intermediates [6]. The reduction step was accomplished on a 1 g and 5 g scale isolating ACBT **8** by filtration through a short silica plug to give the desired product in 60–71% yield. Pure product **8** was recrystallised from hot ethanol for melting point determination.

## Conclusion

We have developed a straight-forward, practical and readily scalable synthesis of ACBT **8**, a valuable intermediate in the development of chemical probes for bioorthogonal ligation and bioluminescent imaging. The procedure allowed the safe synthesis of **8** at multigram scale and in high purity. In addition, the sole use of filtrations and crystallisations for purification of all intermediates and products, in combination with the endothermic nature of the controlled cyanation procedure, will enable straightforward further scale up, if required. A direct comparison of our route to ACBT **8** to those published by others is shown in the Supporting Information File 1, clearly revealing our route as the most suitable for scale-up.

## Experimental

### General experimental

All chemical reagents were purchased from commercial suppliers and used without further purification. The identity and purity of known compounds was confirmed through the comparison of experimentally obtained data to values reported in the literature. ^1^H and ^13^C NMR spectra were recorded in deuterated solvents on a Bruker Avance 500 spectrometer. Chemical shifts are referenced to residual solvent peaks and are quoted in ppm. Coupling constants (*J*) are reported to the nearest 0.1 Hz. Assignment of spectra was based on expected chemical shifts and coupling constants, aided by COSY, HMQC, and HMBC, where appropriate. Calorimetry experiments were carried out using HEL AutoMATE parallel reactors with HEL WinISO 2225 and HEL IQ 1.2.16 software. Temperature was controlled using a Julabo refrigerated/heating circulator (Model FP50-HD) and feed reactants introduced into the reaction using a Harvard syringe pump (model pump 11) connected to a 20 mL disposable syringe with an 8 inch, 16 gauge Luer fitting syringe needle.

### Reaction procedures

#### 2-Chloro-6-nitro-1,3-benzothiazole (**6**) [15,20]


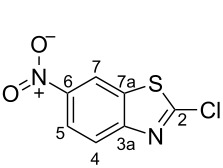


2-Chloro-1,3-benzothiazole (**16**, 10.0 g, 58.95 mmol) was added portion wise to concentrated H_2_SO_4_ (60 mL) in a cooled round-bottomed flask (ice bath). Potassium nitrate (6.56 g, 64.85 mmol) was added portion wise, and the resulting reaction mixture was stirred at 0 °C (ice bath) for 30 min, and then at room temperature for 18 h. The solution was poured onto ice and the formed precipitate collected by filtration. The collected solid was rinsed with ice cold water until acid free, and then dried under reduced pressure. The crude product was further purified by recrystallisation from MeCN (≈ 100 mL) or EtOH (≈ 425 mL) to yield **6** as fine off-white needles. Yield from EtOH (10.37 g, 48.34 mmol, 82%), from MeCN (9.21 g, 42.89 mmol, 73%). Mp 193–194 °C (EtOH); ^1^H NMR (500 MHz, CDCl_3_) δ 8.75 (d, *J* = 2.3 Hz, 1H, C*H-*7), 8.38 (dd, *J* = 9.0, 2.3 Hz, 1H, C*H*-5), 8.07 (d, *J* = 9.0 Hz, 1H, C*H*-4); ^13^C NMR (125 MHz, CDCl_3_) δ 158.9 (*C*-2), 154.9 (*C*-6), 145.6 (*C*-7a), 136.6 (*C*-3a), 123.5 (*C*H-4), 122.4 (*C*H-5), 117.8 (*C*H-7).

#### 6-Nitro-1,3-benzothiazole-2-carbonitrile (**13**) [19]


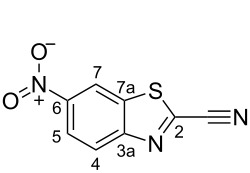


A solution of NaCN (2.40 g, 48.97 mmol) in H_2_O (100 mL) was added slowly to a stirred solution of 2-chloro-6-nitro-1,3-benzothiazole (**6**, 10.0 g, 46.59 mmol) and DABCO (748 mg, 6.99 mmol) in MeCN (1000 mL). The reaction mixture was stirred at room temperature for 24 h. Excess cyanide was quenched by the addition of an aqueous FeCl_3_ solution (0.3 M, 30 mL). The reaction mixture was diluted with H_2_O (470 mL) and extracted with EtOAc (3 × 400 mL). The organic layers were combined, washed with brine (100 mL), dried with Na_2_SO_4_, filtered, and concentrated in vacuo to give a yellow solid. The crude product was loaded onto a short plug of silica gel, flushed through with CHCl_3_ (ca. 1 L) and concentrated in vacuo to give **13** as a white solid (7.89 g, 38.44 mmol, 83%). A sample of **13** was crystallised from MeOH for melting point analysis. *R*_f_ 0.37 (SiO_2_, CHCl_3_); mp 164–165 °C (MeOH); ^1^H NMR (500 MHz, CDCl_3_) δ 8.96 (d, *J* = 2.1 Hz, 1H, C*H-*7), 8.52 (dd, *J* = 9.1, 2.1 Hz, 1H, C*H-*5), 8.38 (d, *J* = 9.1 Hz, 1H, C*H-*4); ^13^C NMR (125 MHz, CDCl_3_) δ 155.5 (*C*-6), 147.4 (*C*-7a), 141.9 (*C*-2), 135.7 (*C*-3a), 126.2 (*C*H-4), 123.2 (*C*H-5), 118.7 (*C*H-7), 112.1 (*C*≡N).

#### 6-Amino-1,3-benzothiazole-2-carbonitrile (**8**) [21]


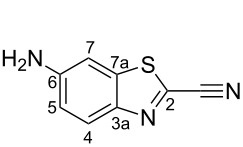


Iron powder (68.05 g, 1218.55 mmol) was added to a suspension of 6-nitro-1,3-benzothiazole-2-carbonitrile (**13**, 5.00 g, 24.37 mmol) in acetic acid (500 mL). The reaction mixture was stirred at room temperature for 24 h, diluted with water (1 L) and unreacted iron was removed by filtration through celite. The aqueous solution was extracted with EtOAc (4 × 500 mL). The combined organic layers were washed with brine (2 × 300 mL), dried (Na_2_SO_4_) and concentrated in vacuo. The crude product was loaded onto a short plug of silica gel, flushed through with CHCl_3_ (ca. 1 L) and concentrated in vacuo to give **8** as yellow microcrystals (3.02 g, 17.24 mmol, 71%). A sample of **13** was crystallised from EtOH for melting point analysis. *R*_f_ 0.2 (SiO_2_, DCM); mp 219–220 °C (EtOH); ^1^H NMR (500 MHz, CDCl_3_) δ 7.95 (d, *J* = 8.9 Hz, 1H, C*H*-4), 7.08 (d, *J* = 2.2 Hz, 1H, C*H-7*), 6.95 (dd, *J* = 8.8, 2.2 Hz, 1H, C*H-*5), 4.14 (s, 2H, N*H_2_*); ^13^C NMR (125 MHz, CDCl_3_) δ 147.8 (*C*-6), 145.7 (*C*-7a), 138.3 (*C*-3a), 131.2(*C*-2), 126.1 (*C*H-4), 117.8 (*C*H-5), 113.7 (*C*≡N), 104.0 (*C*H-7).

## Supporting Information

File 1 Copies of ^1^H and ^13^C NMR spectra, details of calorimetry experiments and comparison of routes to ACBT **8**.
