# Supplementary material for: Economical and scalable synthesis of 6-amino-2-cyanobenzothiazole
Source: Beilstein J Org Chem. 2016 Sep 13;12:2019–25. doi: 10.3762/bjoc.12.189 (PMC5082452; doi:10.3762/bjoc.12.189)
Supplement: File 1 — Copies of 1H and 13C NMR spectra, details of calorimetry experiments and comparison of routes to ACBT 8. [file Beilstein_J_Org_Chem-12-2019-s001.pdf]

Supporting Information

for

Economical and scalable synthesis of 6-amino-2-cyanobenzothiazole

Jacob R. Hauser[1,2], Hester A. Beard[1,2], Mary E. Bayana[1,3], Katherine E. Jolley[1,3], Stuart L. Warriner[1,2], and Robin S. Bon*[2,4]

Address: [1]School of Chemistry, University of Leeds, [2]Astbury Centre for Structural Molecular Biology, [3]Institute of Process Research and Development, and [4]Leeds Institute of Cardiovascular and Metabolic Medicine, LIGHT Laboratories, University of Leeds, Leeds LS2 9JT, UK

Email: Dr Robin S. Bon - r.bon@leeds.ac.uk

* Corresponding author

Copies of ^{1}H and ^{13}C NMR spectra, details of calorimetry experiments and comparison of routes to ACBT 8

Contents

1. Spectral Data S3
   1.1 2-Chloro-6-nitro-1,3-benzothiazole (**6**) S3
   1.2 6-Nitro-1,3-benzothiazole-2-carbonitrile (**13**) S4
   1.3 6-Amino-1,3-benzothiazole-2-carbonitrile (**8**) S5
   1.4 Crude mixture from cyanation reaction in ethanol S6
2. Calorimetry S7
   2.1 Calorimetry Procedures S7
      2.1.1 Addition of NaCN (aq) to 6-nitro-1,3-benzothiazole-2-carbonitrile (**13**) and DABCO in MeCN. S7
      2.1.2 Addition of water to 6-nitro-1,3-benzothiazole-2-carbonitrile (**13**) and DABCO in MeCN S9
   2.2 Calorimetric Data S10
      2.2.1 Addition of NaCN (aq) to 6-nitro-1,3-benzothiazole-2-carbonitrile (**13**) and DABCO in MeCN S10
      2.2.2 Addition of water to 6-nitro-1,3-benzothiazole-2-carbonitrile **13** and DABCO in MeCN S12
      2.2.3 Calculations S14
3. Comparison of routes to ACBT **8** S15

1. Spectral Data

1.1 2-Chloro-6-nitro-1,3-benzothiazole (**6**)

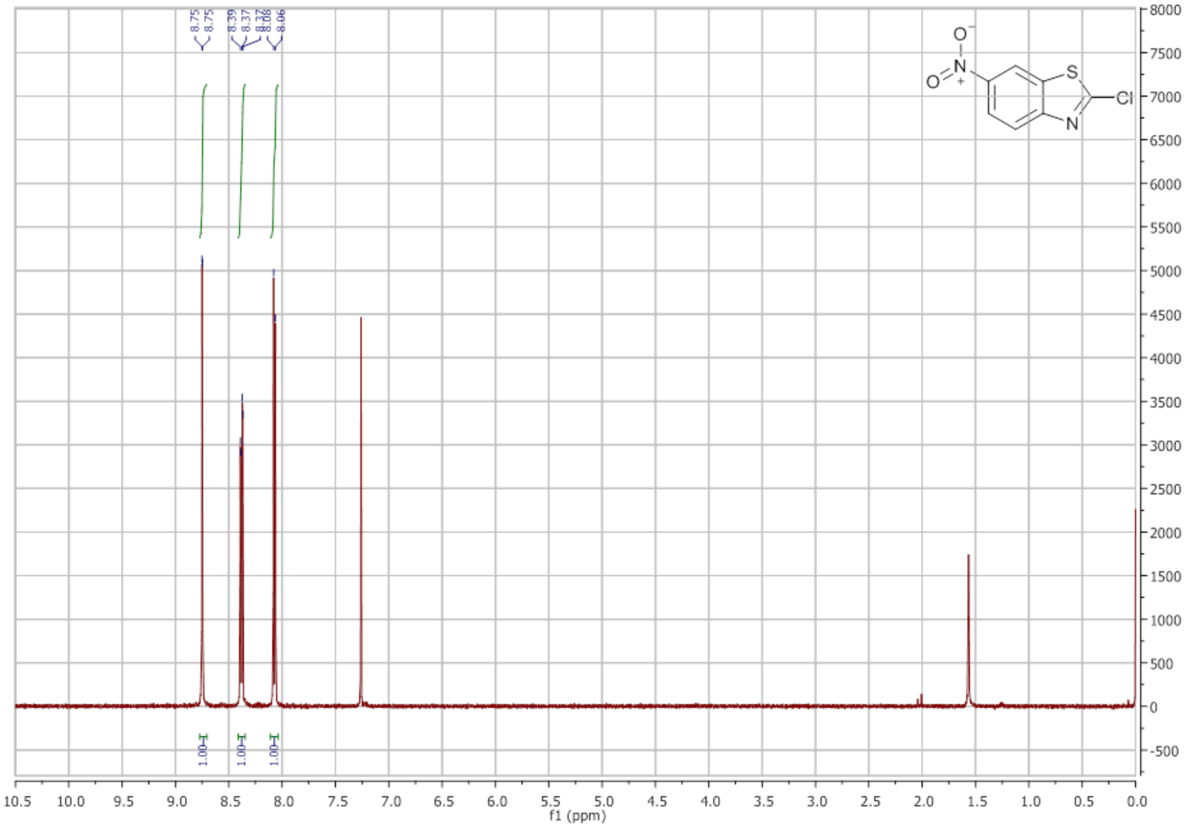

Figure S1: ^{1}H NMR spectrum of 2-chloro-6-nitro-1,3-benzothiazole **6**

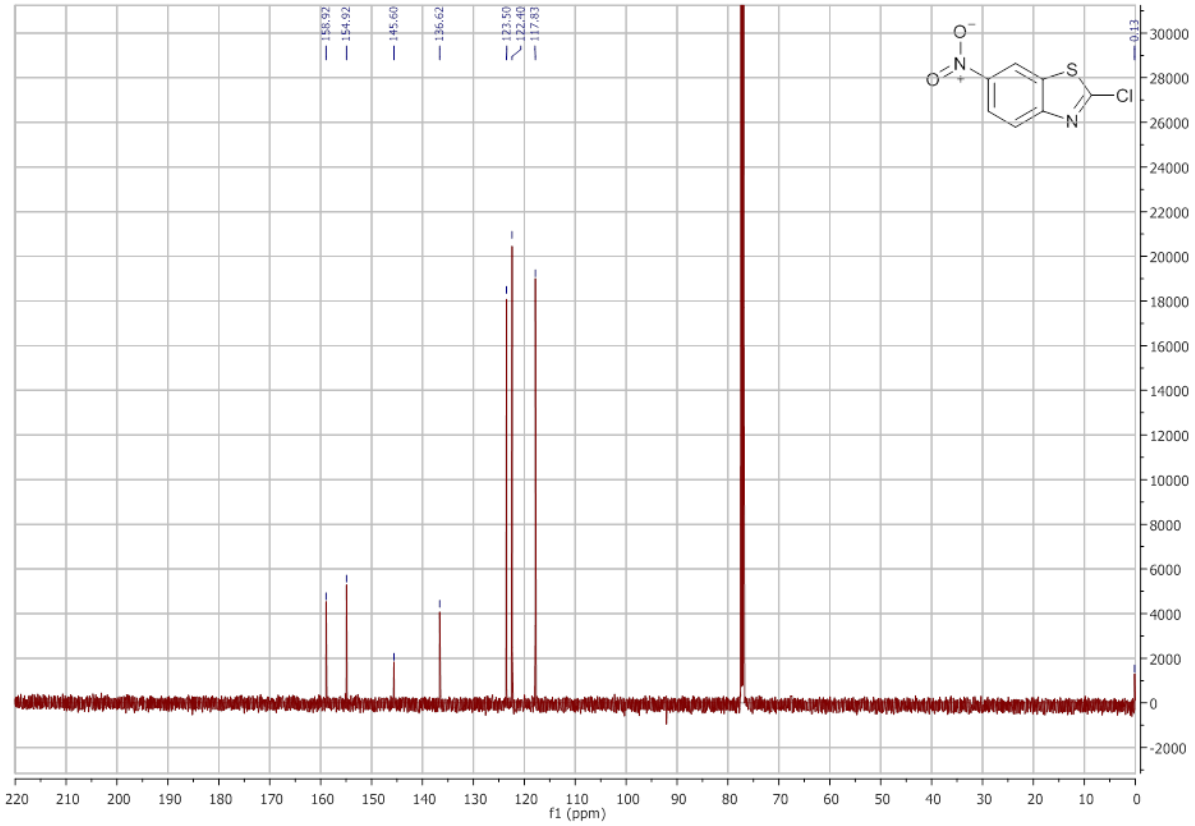

Figure S2: ^{13}C NMR spectrum of 2-chloro-6-nitro-1,3-benzothiazole **6**

1.2 6-Nitro-1,3-benzothiazole-2-carbonitrile (**13**)

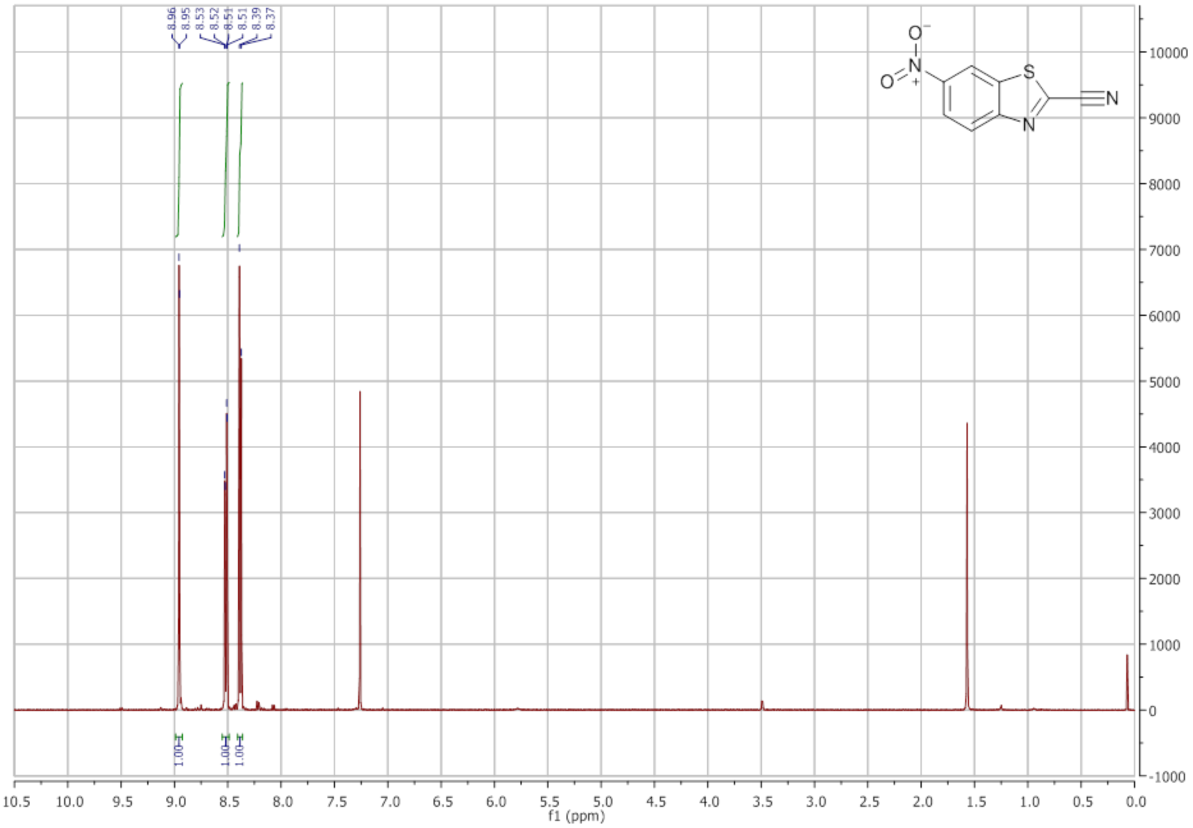

Figure S3: ^{1}H NMR spectrum of 6-nitro-1,3-benzothiazole-2-carbonitrile **13**

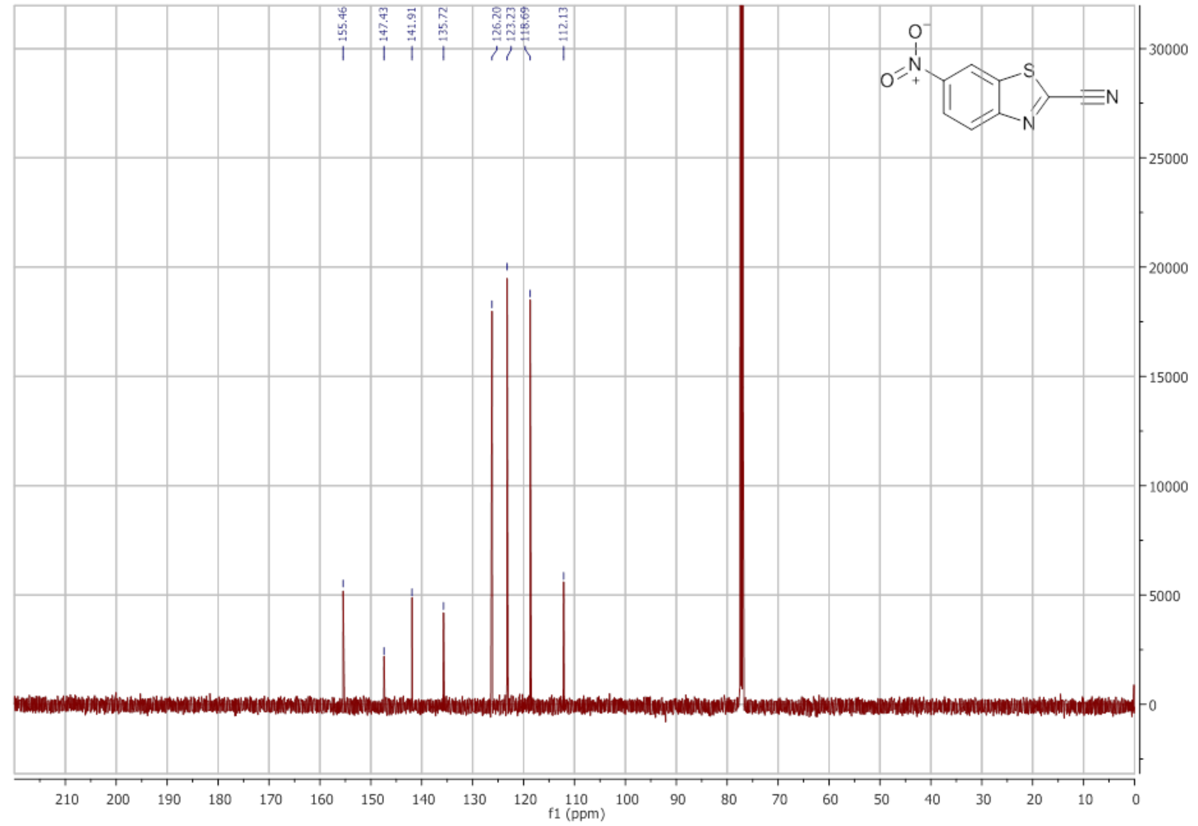

Figure S4: ^{13}C NMR spectrum of 6-nitro-1,3-benzothiazole-2-carbonitrile **13**

1.3 6-Amino-1,3-benzothiazole-2-carbonitrile (**8**)

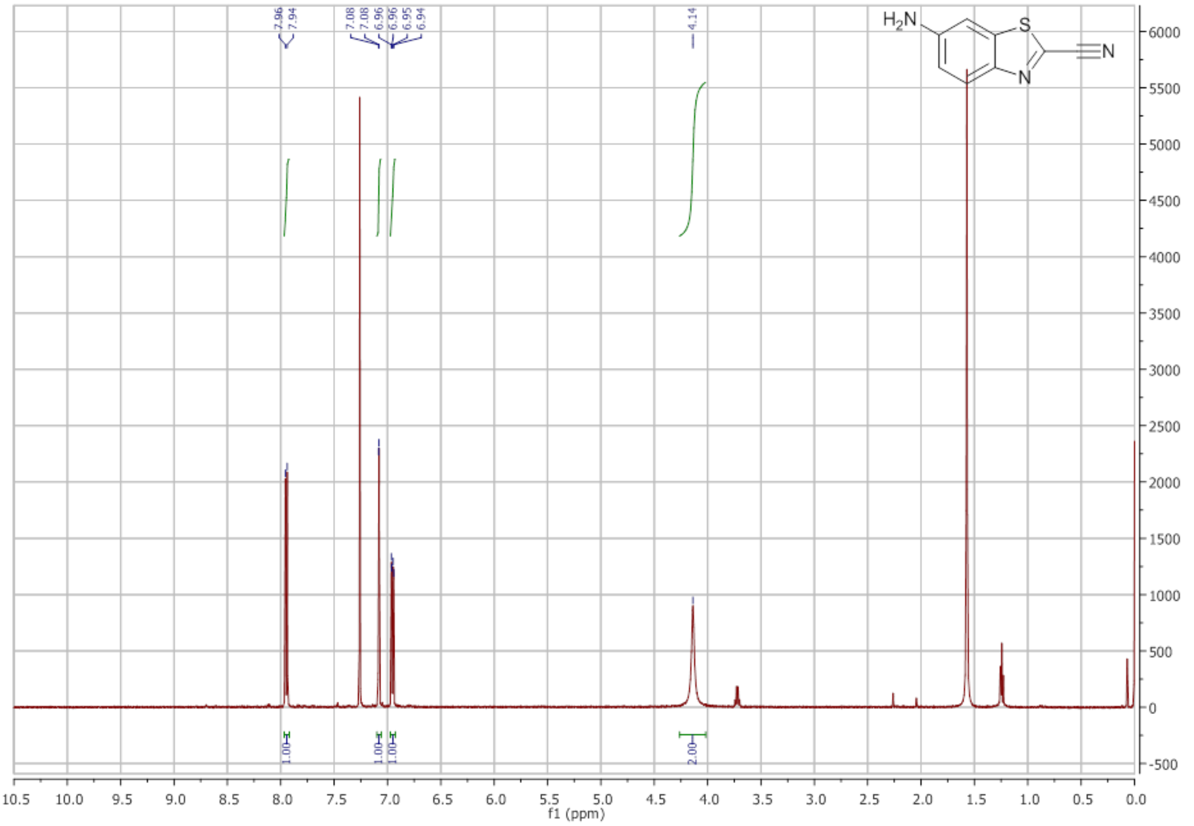

Figure S5*:* ^{1}H NMR spectrum of 6-amino-1,3-benzothiazole-2-carbonitrile **8**

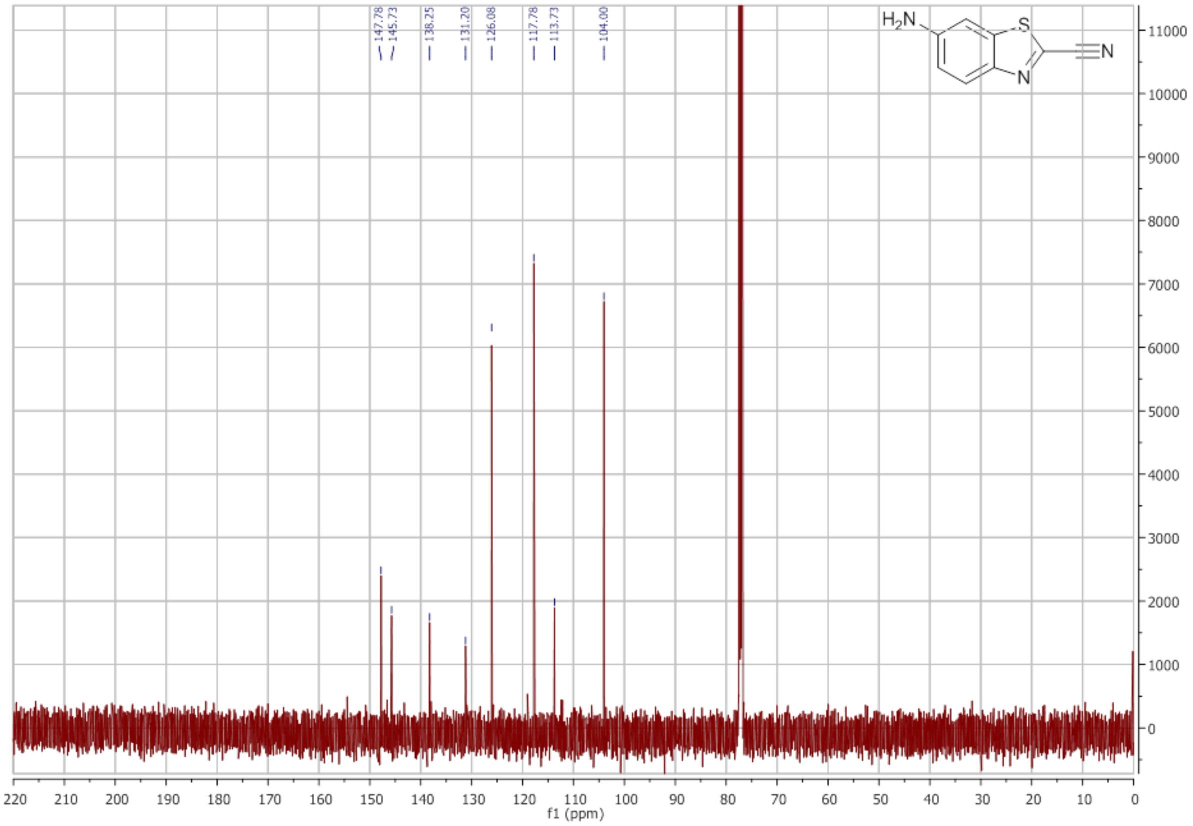

Figure S6*:* ^{13}C NMR spectrum of 6-amino-1,3-benzothiazole-2-carbonitrile **8**

1.4 Crude mixture from cyanation reaction in ethanol

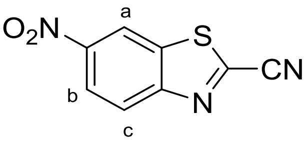

13

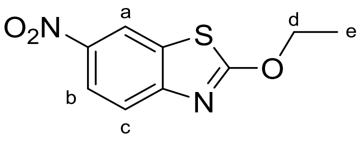

14

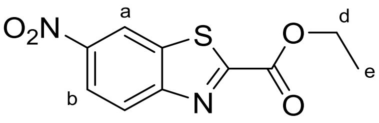

15

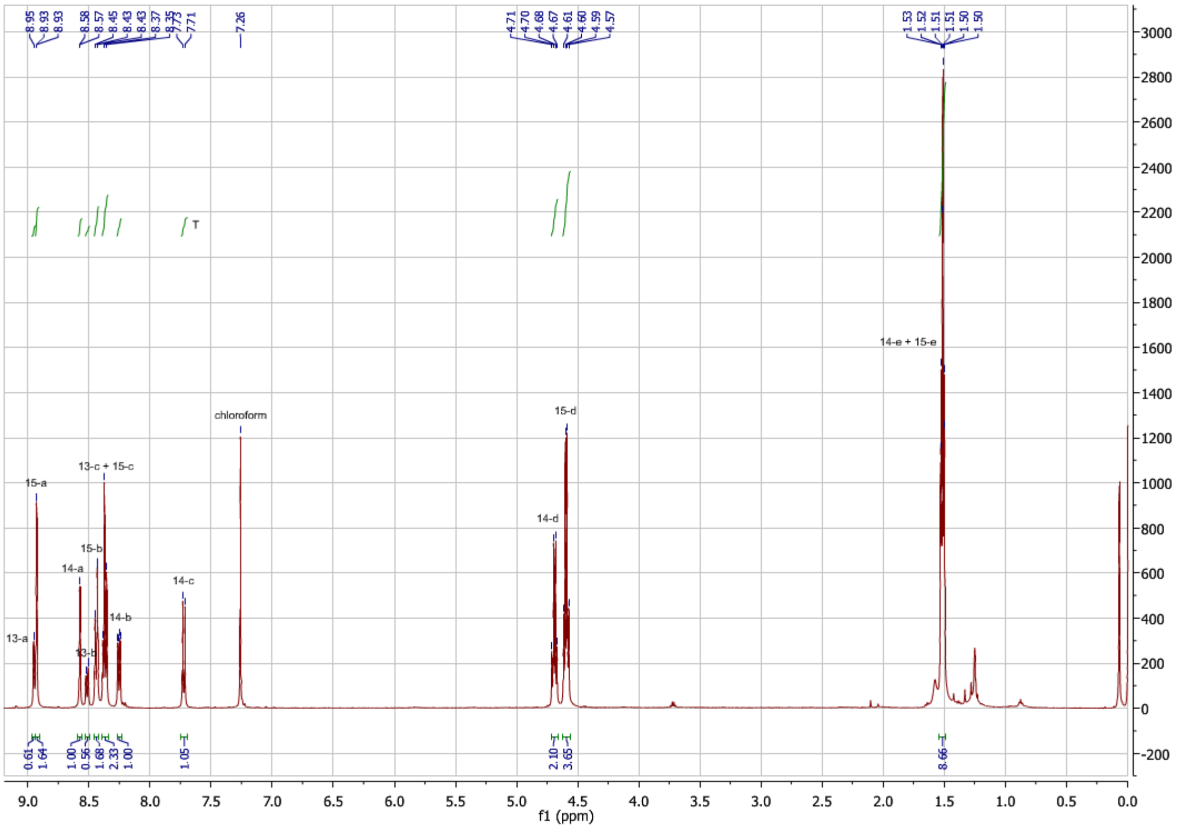

Figure S7: ^{1}H NMR spectrum of a mixture of 6-nitro-1,3-benzothiazole-2-carbonitrile (**13**), 2-ethoxy-6-nitro-1,3-cyanobenzothiazole (**14**) and 6-nitro-2-benzothiazolecarboxylic acid ethyl ester (**15**). The assignment of the three components of the mixture was supported by LC–ESIMS analysis. The ^{1}H NMR peaks for **13** match those in Figure S3, and the ^{1}H NMR peaks for **14** match literature data as described in: Barlin, G.B. and Ireland, S.J. Heterocyclic Amplifiers of Phleomycin. IX* Some Derivatives of Fused and Unfused Mono- and Diaza Heterocycles. *Aust. J. Chem.* **1985**, *38*, 1685–1691.

2. Calorimetry

2.1 Calorimetry Procedures

2.1.1 Addition of NaCN (aq) to 6-nitro-1,3-benzothiazole-2-carbonitrile (**13**) and DABCO in MeCN.

In a 50 mL vessel, 6-nitro-1,3-benzothiazole-2-carbonitrile (**13**, 200 mg, 0.932 mmol) and DABCO (15.7 mg, 0.14 mmol) were dissolved in MeCN (20.0 mL). Aqueous NaCN (0.49 M) was then fed into the reaction as described below, and power compensation calorimetry was used to determine heat changes.

Steady State 1 :

Stir	300 rpm
Reactor Temperature	21 °C
Circulator Temperature	1 °C
Time	60 min

Feed 1 :

Stir	300 rpm
Reactor Temperature	21 °C
Circulator Temperature	1 °C
Feed	0.49 M NaCN (aq) at 0.2 mL min^{-1} for 10 min (2.00 mL, 0.978 mmol NaCN)
Time	10 min

Steady State 2:

Stir	300 rpm
Reactor Temperature	21 °C
Circulator Temperature	1 °C
Time	1080 min

Feed 2 :

Stir	300 rpm
Reactor Temperature	21 °C
Circulator Temperature	1 °C
Feed	0.003 M $FeCl_3$ (aq) at 2 mL min^{-1} for 10 min (20.0 mL, 0.060 mmol $FeCl_3$)
Time	10 min

Steady State 3 :

Stir	300 rpm
Reactor Temperature	21 °C
Circulator Temperature	1 °C
Time	60 min

The IQ software requires input of the specific heat capacity of the feed for the calculation of compensatory power. For this, the specific heat capacity of water (4.18 J g^{-1} K^{-1}) was used.

2.1.2 Addition of water to 6-nitro-1,3-benzothiazole-2-carbonitrile (**13**) and DABCO in MeCN

In a 50 mL vessel, 6-nitro-1,3-benzothiazole-2-carbonitrile (**13**, 200 mg, 0.932 mmol) and DABCO (15.7 mg, 0.14 mmol) were dissolved in MeCN (20.0 mL). Water (2.0 mL) was then fed into the reaction as described below, and power compensation calorimetry was used to determine heat changes.

Steady State 1 :

Stir	300 rpm
Reactor Temperature	21 °C
Circulator Temperature	1 °C
Time	90 min

Feed 1 :

Stir	300 rpm
Reactor Temperature	21 °C
Circulator Temperature	1 °C
Feed	Water at 0.2 mL min^{-1} for 10 min (2.00 mL)
Time	10 min

Steady State 2:

Stir	300 rpm
Reactor Temperature	21 °C
Circulator Temperature	1 °C
Time	50 min

The software requires input of the specific heat capacity of the feed for the calculation of compensatory power. For this, the specific heat capacity of water (4.18 J g^{-1} K^{-1}) was used.

2.2 Calorimetric Data

2.2.1 Addition of NaCN (aq) to 6-nitro-1,3-benzothiazole-2-carbonitrile (**13**) and DABCO in MeCN.

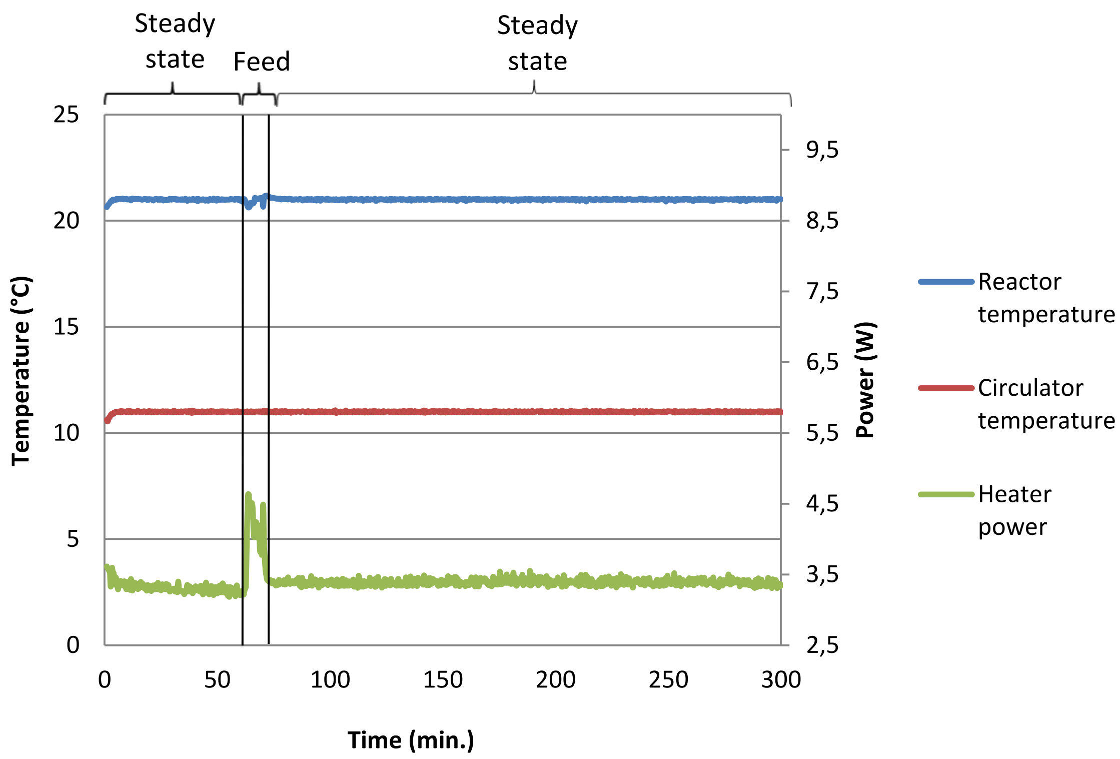

The raw calorimetry data show an increase in the power demand of the internal heater upon addition of the aqueous NaCN solution. This indicates an endothermic process in the reactor requiring greater heating power from the heater to maintain the set reactor temperature of 21 °C.

The IQ software integrates the power output (W) during the reaction to give the total energy (Q_{Total} in kJ) using the equation:

$$Q_{Total} = Q_{Comp} - Q_{Dose}$$

$$Q_{Dose} = m \times C_p \times (T_f - T_i)$$

Where
Q_{Comp} = Heat compensated for by internal heater i.e. the inverse of the heat produced by the reaction
Q_{Dose} = Heat added due to feed being at a different temperature to the reaction temperature
m = total mass of feed (g s^{-1})
C_p = specific heat capacity of feed solution (J g^{-1} °C^{-1})
T_f = final temperature (°C)

T_i = initial temperature (°C)

SIMULAR Energy Report for File JRH1-010a.dat

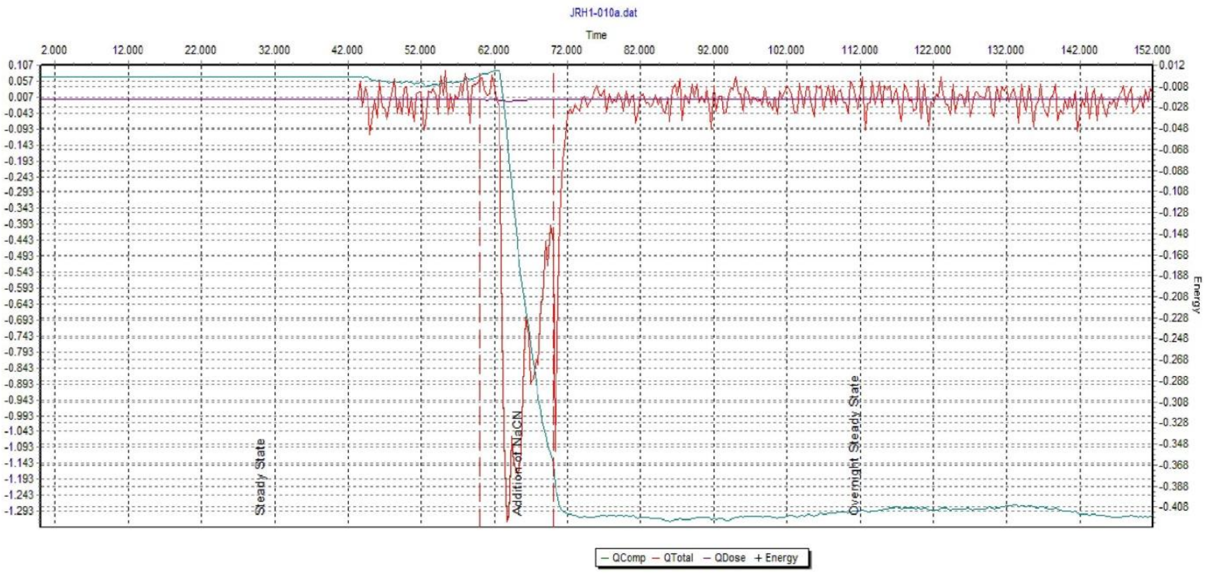

Calculation method = Power Compensation
Baseline Interpolation 1
Start Step = Steady State
End Step = Overnight Steady State
Start Value = 3.27 W
End Value = 3.38 W
Interpolation type : Follow Column Total Flow

REACTION 1
Maximum Total Power = 0.09W
Maximum Reaction Power = 0.09W
Maximum Dose Power = 0.00W

Total Flow/Balance Energy = -0.42kJ
Total Dose Energy = 0.00kJ
=========> Total Energy of -0.42kJ

Total Energy while Dosing = -0.37kJ
Total Energy after Dosing = -0.05kJ

It should be noted that the calculated energies given relate to the output of the heater coil rather than the reaction, i.e. a negative value indicates release of energy from the heater coil and consumption of that energy by the reaction (endothermic reaction). The signs of the energy is therefore inverted to give the true value for the reaction.

There is a very short delay upon the start of the feed before the endotherm is observed. Following the end of the feed there is a further spike, which is due to manual feeding of the remaining aqueous NaCN solution from the syringe. According to these data, 0.37 kJ is consumed during the feed and 0.05 kJ is consumed upon addition of the remaining solution from the syringe, giving a total energy of 0.42 kJ.

2.2.2 Addition of water to 6-nitro-1,3-benzothiazole-2-carbonitrile **13** and DABCO in MeCN.

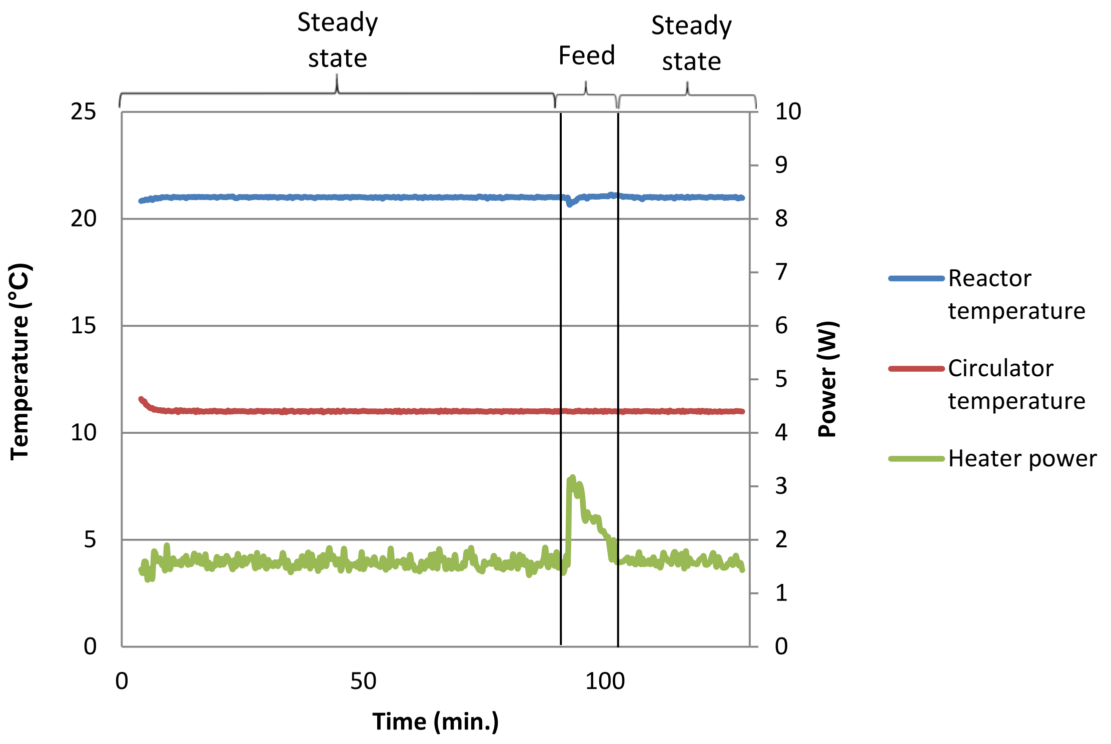

The raw calorimetry data show an increase in the power demand of the internal heater upon addition of water. This indicates an endothermic process in the reactor requiring greater heating power from the heater to maintain the set reactor temperature of 21 °C.

SIMULAR Energy Report for File control.dat

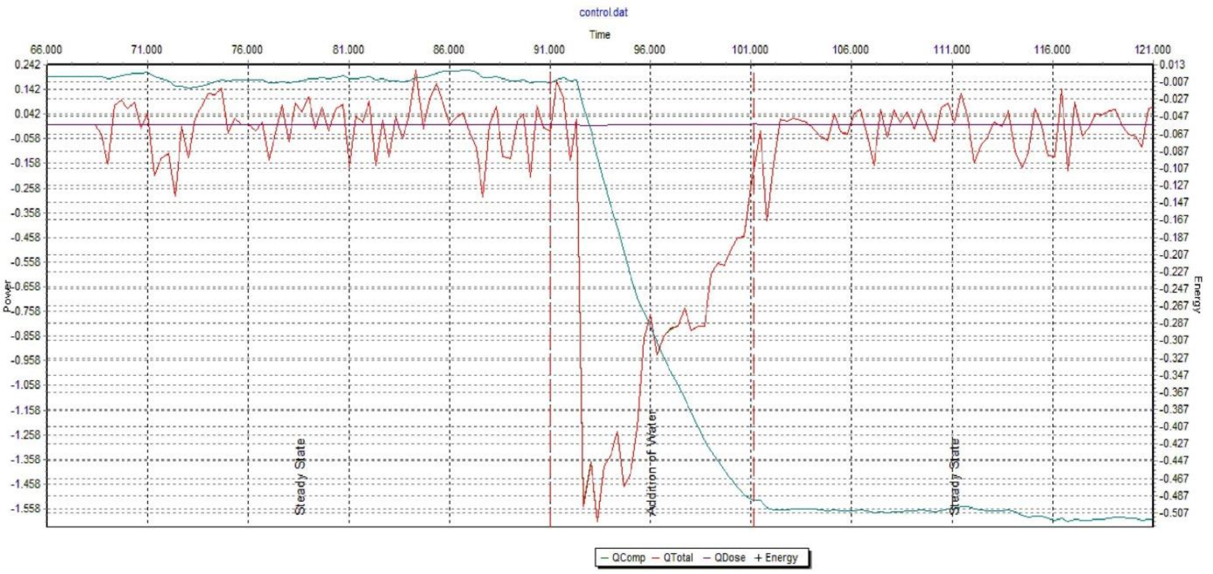

Calculation method = Power Compensation
Baseline Interpolation 1
Start Step = Steady State
End Step = Steady State
Start Value = 1.56 W
End Value = 1.60 W
Interpolation type : Follow Column Total Flow

REACTION 1
Maximum Total Power = 0.22W
Maximum Reaction Power = 0.22W
Maximum Dose Power = 0.00W

Total Flow/Balance Energy = -0.51kJ
Total Dose Energy = 0.00kJ
=========> Total Energy of -0.51kJ

Total Energy while Dosing = -0.49kJ
Total Energy after Dosing = -0.02kJ

Molar Heat = -4.57kJ based on dose of 2.00g

It should be noted that the calculated energies and molar heat given relate to the output of the heater coil rather than the reaction, i.e. a negative value indicates release of energy from the heater coil and consumption of that energy by the reaction (endothermic reaction). The signs of the energy and molar heat values are therefore inverted to give the true value for the reaction.

There is a short delay upon the start of the feed before the endotherm is observed. Following the end of the feed there is a further spike, which is due to manual feeding of the remaining water in the syringe. According to these data, 0.49 kJ is consumed during the feed and 0.02 kJ is consumed upon addition of the remaining water from the syringe, giving a total energy of 0.51 kJ.

The calculated molar heat in this experiment (ΔH = +4.57 kJ mol^{-1}) is the energy per mole water added.

2.2.3 Calculations

Molar heat calculation for addition of NaCN to the reaction mixture:

$$E_{(NaCN)} = E_{(NaCN+water)} - E_{(water)} = 0.42 - 0.51 = -0.09\ kJ$$

$$\Delta H_{(NaCN)} = E_{(NaCN)} \div n_{(NaCN)} = -0.09 \div 0.001 = -90\ kJ\ mol^{-1}$$

Where:
$E_{(NaCN)}$ = Total energy released from addition of NaCN (calculated)
$E_{(NaCN + water)}$ = Total energy released from addition of NaCN in water (0.5 M) (measured)
$E_{(water)}$ = Total energy released from addition of water (measured)
$\Delta H_{(NaCN)}$ = Heat of reaction for the addition of 1 mole of NaCN (calculated)
$n_{(NaCN)}$ = Moles of NaCN added to reaction (measured)

It is important to note that this calculation of molar heat of reaction is an approximation, as the properties (specific heat capacity, ionic strength) of the feeds in the reaction and control experiment are different.

3. Comparison of routes to ACBT **8**

A: Takakura et al. 2011 and Wang et al. 2013

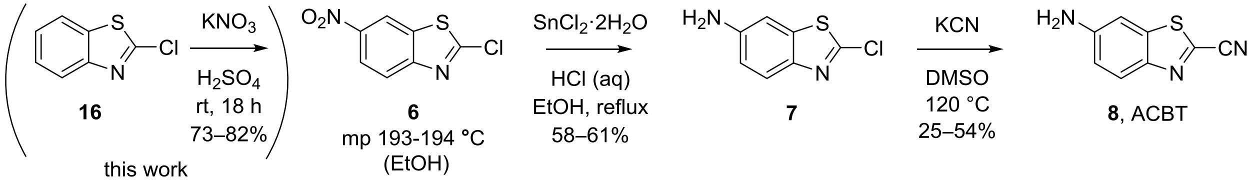

B: McCutcheon et al. 2015

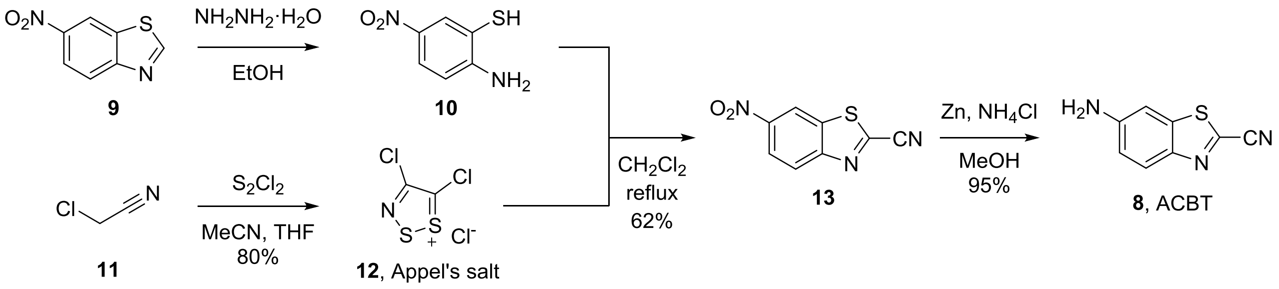

C: This work

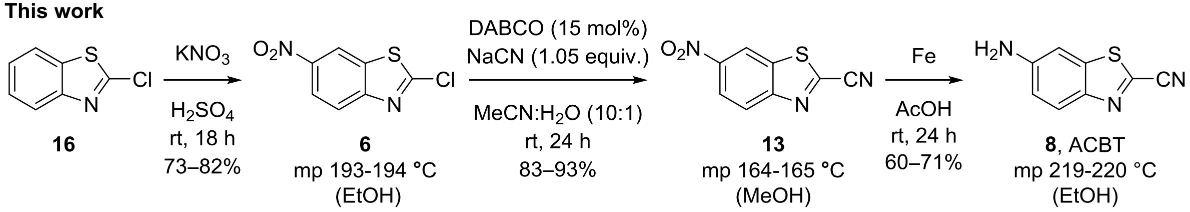

Table S1: Comparison of routes to ACBT **8**

Route	Starting material(s)	Number of steps	Purification steps	Reported overall yield	Estimated costs of reagents to produce 10g of ACBT **8** [a]	Comments
A	**6**	2	**6** → **7**: extraction and column<br>**7** → **8**: (extensive) extraction and column	15–33%	**6**: £751-£1706<br>**$SnCl_2$*H_2O**: £37-£85<br>**Total**: £788-£1791	Dilute conditions of cyanation of **7** cause significant problems during work-up. This, together with the danger of using KCN in DMSO at high temperature, make this reaction unsuitable for scale-up beyond ca. 1g scale (leading to only ca. 500 mg of **8**) in a typical research lab.
	**16** [b]	3	**16** → **6**: precipitation and crystallisation<br>**6** → **7**: extraction and column<br>**7** → **8**: (extensive) extraction and column	6–27%	**16:** £30-£80<br>**KNO_3**: <£1<br>**$SnCl_2$*H_2O**: £36-£85<br>**Total**: £67-£166	
B	**9** and **12**	3	**9** → **10**: precipitation<br>**10** + **12** → **13**: column<br>**13** → **8**: filtration and column	59%	**12:** £808<br>**9**: £69<br>**Hydrazine**: £12<br>**Zinc powder**: £5<br>**Total**: £894	In all our attempts, reduction of **13** with zinc powder partially stalled at the corresponding nitroso intermediate; the source/particle size of zinc powder may be critical.<br><br>Handling of highly toxic sulfur monochloride requires elaborate safety measures.
	**9** and **11**	4	**9** → **10**: precipitation<br>**11** → **12**: precipitation<br>**10** + **12** → **13**: column<br>**13** → **8**: filtration and column	47%	**11:** £1<br>**S_2Cl_2:** £1<br>**9**: £69<br>**Hydrazine**: £12<br>**Zinc powder**: £5<br>**Total**: £88	
C	**6**	2	**6** → **13**: extraction and filtration<br>**13** → **8**: extraction and filtration	50–66%	**6:** £375-£497<br>**Iron powder**: £14-£19<br>**DABCO**: <£1<br>**Total**: £390-£516	For the DABCO-catalysed cyanation of **6**, the use of MeCN as solvent, slow addition of aqueous NaCN (to prevent an exotherm), and quench of unreacted cyanide with aqueous $FeCl_3$ make this step more appropriate for scale-up. In our hands, the reduction of **13** with iron powder was more straight-forward than reduction with zinc powder (as described by McCutcheon et al.)
	**16**	3	**16** → **6**: precipitation and crystallisation<br>**6** → **13**: extraction and filtration<br>**13** → **8**: extraction and filtration	37–54%	**16:** £16-£23<br>**KNO_3**: <£1<br>**Iron powder**: £14-£19<br>**DABCO**: <£1<br>**Total**: £32-£44	

[a]Costs of starting materials, reagents and catalysts were estimated based on the best catalogue prices listed on ScienceWarehouse on 3 August 2016; costs of bulk chemicals (sulfuric acid, HCl (aq), $FeCl_3$, KCN, NaCN), solvents and consumables required for purifications have not been included. [b]Calculations for **16** → **6** were based on reaction/purification conditions and yield described in this work.
